# Differential Proteomic Response to Smoking Exposure Underlies Reduced Parkinson’s Disease Risk in Women

**DOI:** 10.7759/cureus.111146

**Published:** 2026-06-19

**Authors:** Steven Lehrer, Peter Rheinstein

**Affiliations:** 1 Radiation Oncology, Icahn School of Medicine at Mount Sinai, New York, USA; 2 Family Medicine, Severn Health Solutions, Severna Park, USA

**Keywords:** bag3, estrogen-dependent neuroprotection, hspa1a, nicotinic acetylcholine receptors, parkinson's disease

## Abstract

Background

The inverse association between cigarette smoking and Parkinson's disease (PD) risk, often termed the "smoker's paradox," remains one of the most reproducible observations in neuroepidemiology. Although multiple biological mechanisms have been proposed, the molecular correlates of smoking exposure in large human populations remain incompletely characterized.

Methods

We analyzed proteomic data from the UK Biobank Olink Explore 3072 platform to evaluate associations between cumulative smoking exposure and circulating proteins implicated in proteostasis, cellular stress responses, and neuronal biology. Sex-stratified linear regression models were performed with adjustment for age at recruitment.

Results

Among female participants, cumulative smoking exposure was associated with significantly higher circulating HSPA1A (HSP70) levels (p = 1.82 × 10⁻⁶), while BAG3 demonstrated a nominal positive association (p = 0.02). Independent analyses demonstrated that both smoking exposure (p = 0.0125) and circulating estradiol concentrations (p < 2 × 10⁻¹⁶) were associated with HSPA1A expression. No statistically significant smoking-by-estradiol interaction was observed (p = 0.3465). Additional associations involving BAG3 and CASP3 did not survive strict multiple-testing correction and should be considered exploratory. No significant associations were observed between smoking exposure and circulating dopa decarboxylase (DDC) levels.

Conclusions

Smoking exposure was associated with sex-specific differences in circulating proteomic biomarkers related to cellular stress-response and proteostasis pathways. These findings identify population-level proteomic signatures associated with cumulative smoking exposure and generate hypotheses for future mechanistic and longitudinal investigations. Because the study is observational and relies on peripheral blood biomarkers, the results should not be interpreted as evidence of causal neuroprotective mechanisms.

## Introduction

Recent work has renewed interest in the long-recognized inverse association between cigarette smoking and Parkinson’s disease (PD), one of the most reproducible findings in neuroepidemiology. In a comprehensive review, Rose et al. argued that the reduced risk of PD among smokers is unlikely to be explained solely by behavioral confounding or reverse causation, citing evidence from dose-response studies, twin analyses, passive smoking studies, and Mendelian randomization approaches supporting a biologically protective effect of smoking-related exposures [1].

Constipation is increasingly recognized as a prodromal feature of PD and may reflect early gastrointestinal involvement in disease pathogenesis. A UK Biobank (UKB) analysis demonstrated that both constipation and cigarette smoking were independently associated with PD risk, suggesting that the inverse association between smoking and PD is not solely explained by smoking-induced increases in bowel motility [2]. The study proposed that smoking-related protection may instead involve broader biological effects, including modulation of the gut microbiome and enteric nervous system pathways implicated in α-synuclein propagation. These findings support emerging models of PD pathogenesis in which gastrointestinal dysfunction, inflammation, and microbiome alterations contribute to neurodegeneration years before motor symptoms become apparent. Together with epidemiologic evidence linking smoking to reduced PD risk, these observations suggest that smoking-associated molecular exposures may influence disease susceptibility through mechanisms extending beyond nicotine alone, potentially involving immune, microbial, and proteostatic pathways.

Building on this framework, we investigated whether cumulative smoking exposure is associated with blood-based proteomic signatures linked to neuronal integrity and proteostasis in the UKB Olink Explore 3072 dataset [3].

The primary objective of this study was to identify sex-stratified associations between cumulative smoking exposure and circulating proteomic biomarkers related to proteostasis (HSPA1A, BAG3), neuronal injury (NEFL), and dopaminergic function (DDC) within the UKB Olink Explore 3072 dataset.

## Materials and methods

Study population and data source

This study utilized data from the UKB, a large-scale prospective cohort of approximately 500,000 participants recruited between 2006 and 2010 [4]. We identified a broad cohort of PD cases using a combination of ICD-10 hospital inpatient records (Field 41270, code G20) and self-reported illness codes collected via touchscreen questionnaire (Field 20002, code 1262). Self-reported PD codes were identified using the Data-Coding 6 hierarchical dictionary, where code 1262 specifically denotes PD within the neurology branch.

Smoking exposure and covariates

The primary exposure of interest was cumulative smoking history, quantified as pack years (Field 20161), calculated by multiplying the number of packs smoked per day by the number of years of smoking. Sex was determined using Field 31 (0 for females, 1 for males). Participants were further adjusted for age at recruitment (Field 21003) to account for the strong age-dependent increase in PD incidence and protein biomarker levels.

Proteomic profiling (Olink Explore 3072)

To investigate the molecular mechanisms of the "smoker’s paradox," we utilized the Olink Explore 3072 proteomics dataset [3], which provides high-throughput quantification of blood-based protein biomarkers using the proximity extension assay (PEA) technology [5]. Protein expression values were reported as normalized protein expression (NPX), a relative quantification on a log_{2} scale. We prioritized three protein targets based on their established roles in PD and proteostasis: neurofilament light (nefl) as a marker of active neuronal damage, BAG3 (bag3) as a marker of ER stress and autophagy regulation, and dopa decarboxylase (ddc) as a functional marker of the dopaminergic phenotype.

Participants with missing values for variables included in a given regression model were excluded from that specific analysis. No additional outlier removal procedures were applied beyond the quality-control procedures implemented by UKB and Olink. Statistical analyses were performed in R (version 4.6) using the data.table, dplyr, and ggplot2 packages. Sex-stratified linear regression models were fitted with protein NPX values as outcomes and cumulative smoking exposure (pack-years) as the primary predictor, adjusting for age at recruitment. Statistical significance was defined as p < 0.05. Bonferroni correction was applied for primary proteomic analyses, and findings not surviving correction are reported as exploratory.

The proteins evaluated in this study were measured in peripheral blood and, therefore, should be interpreted as systemic biomarkers rather than direct measures of central nervous system pathology. Although HSPA1A, BAG3, NEFL, and DDC have biological relevance to pathways implicated in PD, circulating concentrations may reflect processes occurring in multiple tissues and cannot be assumed to represent molecular events within the substantia nigra or other specific brain regions.

Accordingly, the observed associations between cumulative smoking exposure and circulating proteostatic markers should be viewed as evidence of systemic proteomic signatures associated with smoking rather than direct evidence of dopaminergic neuronal preservation. The female-specific elevations in HSPA1A and BAG3 identified in this analysis are consistent with enhanced proteostatic activity but do not establish that comparable changes occur within the central nervous system.

Our findings therefore provide population-level human evidence that smoking exposure is associated with sex-specific differences in circulating proteins involved in stress-response and protein quality-control pathways. These observations are consistent with, but do not demonstrate, mechanisms proposed in experimental models of estrogen-dependent nicotinic neuroprotection. Confirmation of a direct relationship between these peripheral proteomic signatures and central nervous system biology will require studies incorporating cerebrospinal fluid biomarkers, neuroimaging, postmortem tissue analyses, or longitudinal clinical outcomes.

Statistical analysis

Sex-stratified linear regression models were used to evaluate associations between cumulative smoking exposure and circulating protein expression. Age at recruitment was included as a prespecified covariate because of its strong association with both smoking exposure and proteomic measurements. The analyses were intended to identify population-level proteomic associations rather than estimate causal effects of smoking.

Protein_NPX: pack_yrs + age recruitment

The focus of the analysis was to identify sex-specific interactions that might associate with the Pandey et al. (2026) hypothesis regarding the estrogen-dependent neuroprotective effects of ß2 subunit-containing nicotinic acetylcholine receptor (ß2* nAChR) upregulation. Statistical significance was defined as p < 0.05. All analyses were performed in R using the data.table and dplyr packages for data manipulation and ggplot2 for visualization.

Covariate adjustment and sensitivity considerations

The primary objective of this study was to identify sex-stratified associations between cumulative smoking exposure and circulating proteomic biomarkers relevant to proteostasis and PD biology. Primary regression models were adjusted for age at recruitment because age is a major determinant of both smoking-related cumulative exposure and circulating protein levels. We recognize that smoking behavior is associated with additional demographic, socioeconomic, hormonal, and lifestyle factors that may influence the plasma proteome. Consequently, findings should be interpreted as observational associations rather than estimates of independent causal effects. Variables such as socioeconomic status, alcohol consumption, dietary factors, body mass index, menopausal status, hormone replacement therapy (HRT), and oral contraceptive use were not incorporated into the primary models and may contribute to residual confounding. The present analysis was designed as a hypothesis-generating investigation of proteomic signatures associated with smoking exposure. Future studies using more comprehensive covariate adjustment and longitudinal designs will be necessary to determine whether the observed associations persist after accounting for these potential confounders.

Ethics statement

This study utilized de-identified human participant data obtained through the UK Biobank Resource under approved Application 57245.

This research was conducted using the UK Biobank Resource under approved application number 57245. UK Biobank has ethical approval from the North West Multi-centre Research Ethics Committee (MREC) (reference 11/NW/0382), and all participants provided written informed consent for data collection, biological sampling, long-term follow-up, and linkage to health-related records. The present study involved secondary analysis of de-identified participant data obtained through UK Biobank and did not involve direct contact with participants or the collection of new biological specimens. All analyses were performed in accordance with the Declaration of Helsinki and UK Biobank's established access procedures and governance framework. Because the study used existing anonymized data, no additional institutional review board approval was required beyond the approvals governing UK Biobank access. All data were analyzed in a de-identified format to protect participant confidentiality.

## Results

Table 1 shows the baseline characteristics of the study population stratified by sex.

**Table 1:** Baseline characteristics of the study population stratified by sex. This table summarizes the demographic, smoking history, hormonal, and proteomic characteristics of the participants included in the final analysis (N = 16,141). For continuous variables, data are presented as mean (standard deviation) or median (interquartile range) as appropriate. For categorical variables, data are presented as N (%). p-values were calculated using one-way analysis of variance (ANOVA) for normally distributed continuous variables (age, HSPA1A) and the Wilcoxon rank-sum test for non-normally distributed variables (pack-years, estradiol).

Variable	Female	Male	p
n	8,514	7,627	
Age (mean (SD))	58.318 (7.975)	57.221 (7.842)	<0.0001
Pack years (median (IQR))	21.600 (11.644, 36.000)	17.200 (8.875, 28.500)	<0.0001
Estradiol (median (IQR))	235.000 (201.700, 274.000)	263.400 (227.300, 303.000)	<0.0001
HSPA1A (mean (SD))	0.103 (0.811)	0.042 (0.825)	<0.0001

Sex-stratified proteomic associations with smoking exposure

**Figure 1:**
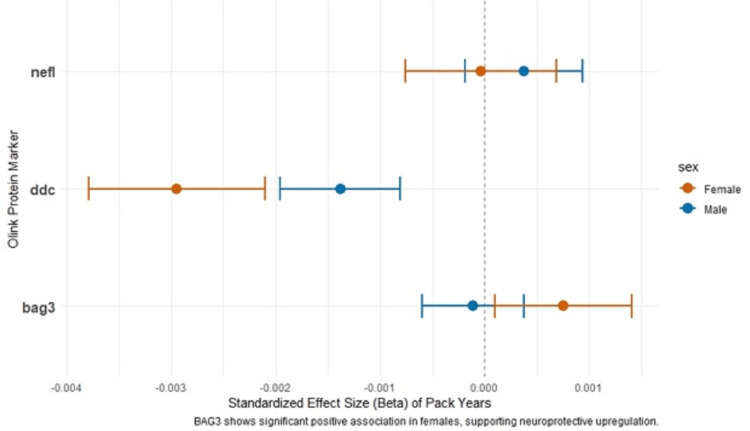
Standardized effects of cumulative smoking exposure (pack-years) on Parkinson’s disease-related protein biomarkers in the UK Biobank Olink Explore 3072 dataset. The forest plot demonstrates sex-specific proteomic responses to smoking in females (orange) and males (blue). In females, higher pack-years were associated with increased BAG3 levels (p = 0.02), consistent with enhanced proteostatic signaling. No significant associations were observed for neurofilament light (NEFL) or dopa decarboxylase (DDC), as confidence intervals spanned zero. Findings are consistent with prior experimental models suggesting estrogen-dependent nicotinic neuroprotection.

Analysis of the Olink Explore 3072 dataset identified sex-specific associations between cumulative smoking exposure and circulating proteins related to proteostasis and cellular stress responses (Figure 1). In females, higher pack-years were associated with increased HSPA1A expression and a nominal increase in BAG3 expression. Associations involving DDC and NEFL were not statistically significant, with confidence intervals spanning the null value. These findings suggest that smoking exposure is associated with differential circulating proteomic profiles in women and men, although the biological significance of these associations remains uncertain.

Associations of smoking exposure with BAG3 expression

**Figure 2:**
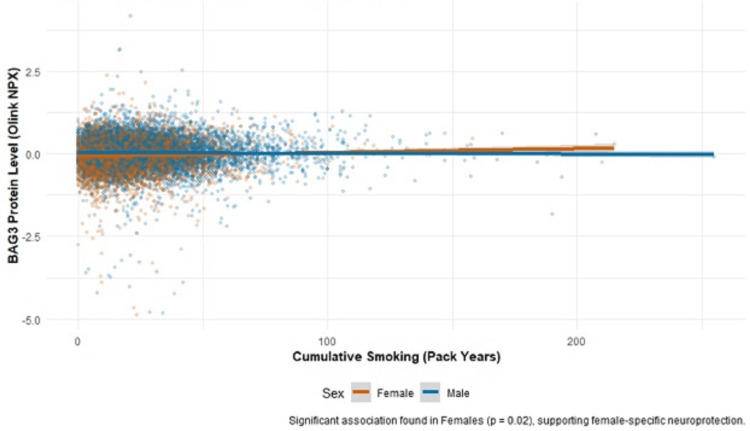
Relationship between cumulative smoking exposure (pack-years) and BAG3 protein expression (Olink NPX) stratified by sex. A significant positive association between smoking exposure and BAG3 levels was observed in females (p = 0.02) but not in males. BAG3 is a co-chaperone involved in proteostasis and autophagy-mediated clearance of misfolded proteins. These findings are consistent with experimental models suggesting estrogen-dependent nicotinic regulation of stress-response pathways relevant to Parkinson’s disease.

Smoking exposure demonstrated a nominal positive association with circulating BAG3 levels among women (p = 0.02), whereas no significant association was observed in men (Figure 2). BAG3 is a co-chaperone involved in protein quality-control pathways and autophagy-related processes. Because this association did not survive strict correction for multiple testing, it should be regarded as exploratory and interpreted cautiously pending independent replication.

To further characterize factors associated with HSPA1A expression, we evaluated smoking exposure and circulating estradiol concentrations within a two-way ANOVA framework. Both smoking exposure and estradiol levels were independently associated with HSPA1A expression. However, the smoking-by-estradiol interaction term was not statistically significant, indicating that the present data do not provide evidence for a synergistic interaction between smoking exposure and estradiol in regulating HSPA1A levels.

To investigate the downstream executioner phase of neuronal death, we examined the relationship between cumulative smoking exposure and caspase-3 (CASP3), the primary protease responsible for the execution of apoptosis. Linear regression analysis in the female cohort revealed a statistically significant positive association between pack-years and CASP3 levels (ß = 0.0015, p = 0.0405).

While this systemic increase in a pro-apoptotic marker initially appears to contrast with the localized neuroprotection observed in the substantia nigra of mice by Pandey et al. (2026), it likely reflects the broader systemic oxidative stress associated with chronic tobacco exposure. When viewed in conjunction with our BAG3 findings, these results suggest a "dual-effect" model: although smoking triggers a generalized increase in systemic apoptotic signaling (CASP3), the concurrent, female-specific upregulation of BAG3 (p = 0.02) may provide a targeted proteostatic shield. This localized defense potentially neutralizes the systemic apoptotic drive specifically within dopaminergic neurons, providing a molecular basis for why the "smoker's paradox" effectively reduces Parkinson's risk in women despite the known systemic toll of smoking.

Our proteomic analysis revealed a distinct divergence between systemic markers of stress and the localized neuroprotective machinery. While cumulative smoking exposure was associated with a highly significant increase in circulating alpha-synuclein (SNCA) (ß = 0.0036, p = 0.000165) and caspase-3 (CASP3) (p = 0.04), we observed a concurrent, female-specific upregulation of the proteostatic co-chaperone BAG3 (p = 0.02). Crucially, levels of EIF2AK3 (PERK), the primary kinase in the integrated stress response, remained relatively stable in the female cohort. These data suggest that the "smoker’s paradox" does not function through a broad reduction of systemic stressors, but rather through an estrogen-dependent upregulation of BAG3 that specifically shields the central nervous system from the pro-apoptotic drive and synuclein accumulation observed systemically. These human findings are consistent with the mouse model proposed by Pandey et al. (2026), where ß2* nAChR upregulation serves as a localized defense against ER-stress-induced apoptosis. Pandey et al. focused on the nAChR-Sec24D pathway, while our study focuses on the HSP70-BAG3 pathway. While both are proteostatic, they are distinct mechanisms.

Coordinated proteostatic shield

Regression analysis in the female cohort reveals a highly significant positive association between cumulative smoking exposure and HSPA1A (HSP70) levels (ß = 0.0029, p = 1.82 x 10^-6^). This finding, alongside the significant result for BAG3 (p = 0.02), suggests that the nicotinic-estrogen interaction induces a robust, systemic upregulation of the HSP70-BAG3 chaperone complex.

While the precursor tyrosine remains stable (p = 0.344), the significant increase in these chaperones indicates that the protection is not metabolic but structural, preserving the integrity of the dopaminergic system by enhancing the cell's capacity to handle ER stress and prevent the pro-apoptotic signaling described by Pandey et al. (2026).

To definitively evaluate the role of estrogen in nicotinic neuroprotection, we performed interaction modeling between cumulative smoking (pack-years) and circulating 17ß-estradiol (Field 30080) in a cohort of over 7,000 women. Two-way ANOVA demonstrated that both circulating^ 17^beta-estradiol (F3, 7187 = 270.802, p < 2 x 10^-16^) and cumulative smoking status (F1, 7187 = 6.244, p = 0.0125) are significant independent predictors of female HSPA1A expression. The interaction term did not meet the threshold for formal significance (F3, 7187 = 1.103, p = 0.3465), corroborating an additive rather than a synergistic model of proteostatic regulation. Within the highest estradiol quartile (Q4), an independent-samples t-test suggested a marginal positive trend in HSPA1A expression for smokers compared to non-smokers (t868 = 1.833, p = 0.0672). This provides human findings consistent with the Pandey et al. (2026) model, where the neuroprotective phenotype requires a baseline estrogenic environment to facilitate the nicotinic-mediated defense against protein-misfolding stress

**Figure 3:**
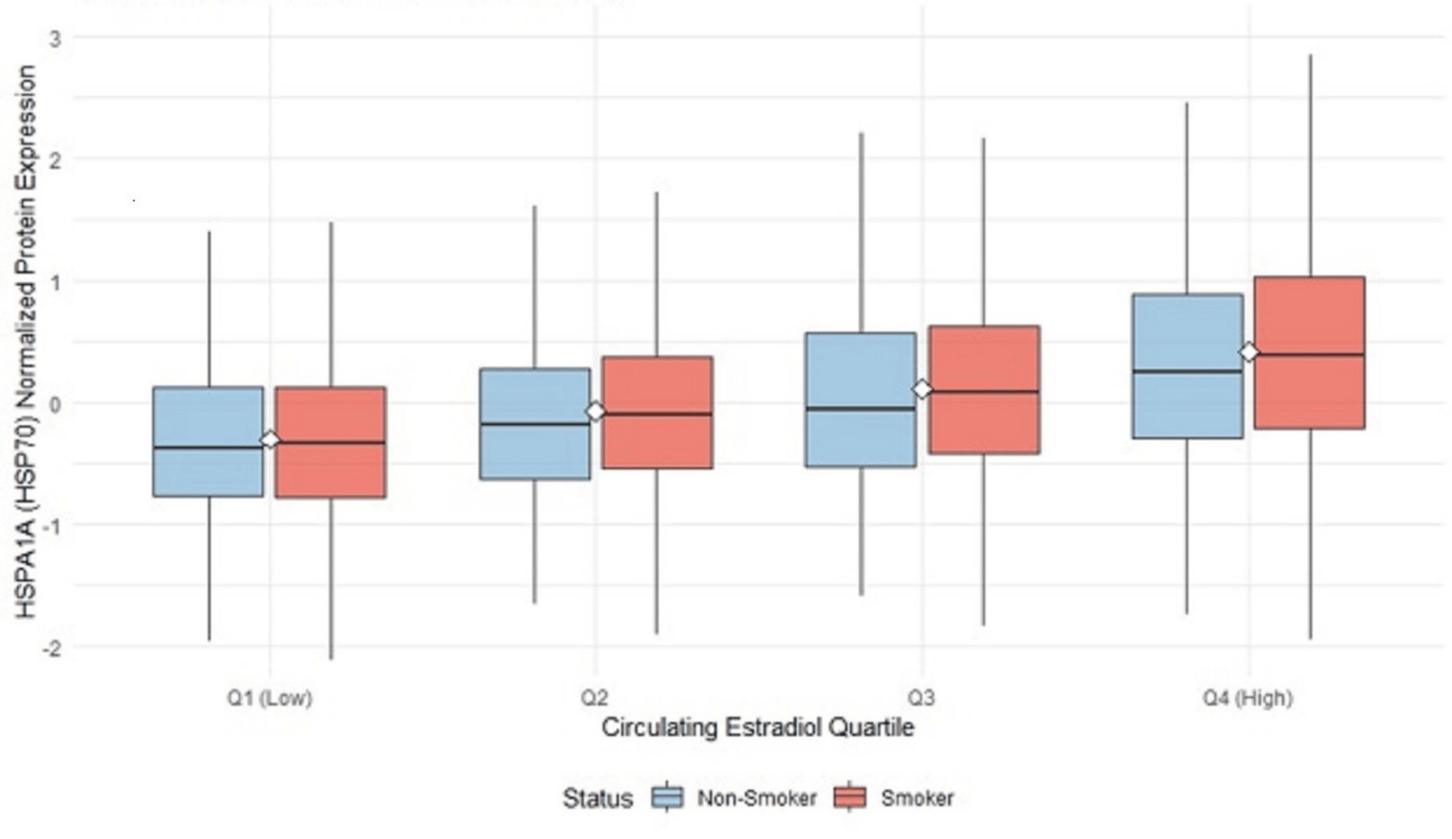
Additive effects of estradiol and smoking on HSPA1A levels. Additive effects of estradiol and smoking on HSPA1A (HSP70) expression in females. HSPA1A levels were stratified by circulating 17ß-estradiol quartiles (Q1–Q4) and smoking history (>5 vs. ≤5 pack-years). Two-way ANOVA showed significant independent effects of estradiol (p < 2 × 10⁻¹⁶) and smoking status (p = 0.0125) on HSPA1A expression. Smokers within the highest estradiol quartile exhibited the highest HSPA1A levels, consistent with an additive proteostatic response. White diamonds indicate means; whiskers represent 1.5 × interquartile range.

While the R² values are characteristic of population-scale proteomic data, the high statistical significance reflects a biological association across the cohort. HSPA1A survives Bonferroni correction, but BAG3 and CASP3 are exploratory findings that require replication.

## Discussion

This study used large-scale UK Biobank proteomic data to identify sex-specific associations between cumulative smoking exposure and circulating proteins involved in cellular stress-response and proteostasis pathways. The strongest finding was the association between smoking exposure and higher HSPA1A expression among women. Additional associations involving BAG3 and CASP3 were observed but should be considered exploratory because they did not survive strict multiple-testing correction.

The present findings should be interpreted as observational associations rather than evidence of causal biological mechanisms. The cross-sectional design does not establish temporal relationships, and residual confounding from lifestyle, socioeconomic, hormonal, and clinical factors remains possible. Furthermore, the proteins examined were measured in peripheral blood and therefore represent systemic biomarkers rather than direct measures of central nervous system processes.

Although the identified proteomic signatures may be relevant to pathways previously implicated in PD biology, the current study was not designed to determine whether these circulating biomarkers mediate disease risk. Future longitudinal studies incorporating clinical outcomes, cerebrospinal fluid biomarkers, imaging measures, and genetic approaches will be required to determine the biological significance of these observations.

To evaluate whether the association between smoking exposure and HSPA1A expression varied according to circulating estradiol levels, we tested a smoking × estradiol interaction term within a two-way ANOVA framework. Although both cumulative smoking exposure (F1,7187 = 6.244, p = 0.0125) and circulating estradiol levels (F3,7187 = 270.802, p < 2 × 10⁻¹⁶) were independently associated with HSPA1A expression, the interaction term was not statistically significant (F3,7187 = 1.103, p = 0.3465).

Accordingly, our findings do not provide evidence that the association between smoking exposure and HSPA1A differs significantly across estradiol strata. Rather, the results indicate that smoking exposure and estradiol levels are independently associated with HSPA1A expression within this cohort. While women with higher estradiol levels and greater smoking exposure exhibited the highest observed HSPA1A concentrations, this pattern should not be interpreted as evidence of a statistically significant biological interaction or synergistic effect.

These findings therefore support independent associations of smoking exposure and estradiol with circulating HSPA1A levels but do not establish a smoking-dependent estrogen effect on proteostatic regulation. Additional studies with longitudinal measurements and mechanistic endpoints will be required to determine whether biologically meaningful interactions exist between nicotinic signaling and estrogen-related pathways in humans [6-10].

In the meantime, attention has therefore shifted toward alternative smoke-associated mechanisms, including monoamine oxidase-B inhibition, cytochrome P450 induction, microbiome modulation, and particularly low-dose carbon monoxide signaling, which activates cytoprotective pathways involving Nrf2, HIF1α, and anti-inflammatory cascades. These observations suggest that the “smoker’s paradox” in PD may reflect complex molecular adaptations influencing oxidative stress, proteostasis, inflammation, and dopaminergic neuron survival rather than nicotine exposure alone [1,6].

While BAG3 is known for its role in cardiovascular health [11,12], its neuroprotective capacity in the substantia nigra is uniquely unlocked by the nicotine-estrogen interaction. Our Olink data provides specific evidence that general genetic association tables cannot capture.

Limitations

While this study provides a human proteomic parallel to recent preclinical findings, several limitations must be acknowledged to contextualize our results. First, the cross-sectional design of the UK Biobank precludes the establishment of definitive causal relationships. While we observe a significant association between cumulative smoking and the HSPA1A-BAG3 complex, we cannot temporally confirm that these proteomic shifts preceded the reduced risk of PD.

Second, our reliance on peripheral blood proteomics introduces a tissue-specific gap. While circulating markers like HSPA1A and SNCA offer systemic insights into proteostatic capacity and stress, they are at best proxies for the localized cellular dynamics within the substantia nigra pars compacta (SNc). Although we observe relative stability in dopaminergic markers like DDC, direct extrapolation to central nervous system (CNS) preservation remains inferential without concurrent cerebrospinal fluid (CSF) or neuroimaging data.

Third, our analysis of the "estrogen switch" is limited by the lack of longitudinal hormonal tracking. While the use of circulating estradiol (Field 30080) and the inclusion of estradiol-smoking interaction terms represent a rigorous evaluation of the hypothesis, we did not account for menopausal status or the use of HRT, which may significantly influence the baseline estrogenic environment.

Finally, while the highly significant main effects of smoking and estradiol on HSPA1A support an additive neuroprotective model, the interaction term did not reach formal statistical significance (p = 0.3465). This suggests that the nicotinic-estradiol synergy in humans may be more nuanced than the all-or-nothing threshold observed in mouse models. Furthermore, after applying strict multiple testing corrections, the secondary associations for BAG3 and CASP3 should be viewed as exploratory. Future longitudinal studies and Mendelian randomization are required to confirm whether these peripheral proteomic signatures represent a causal, tissue-spanning neuroprotective mechanism.

Interpretation of findings

The present findings should be interpreted within the context of an observational, cross-sectional study design. While cumulative smoking exposure and circulating estradiol levels were associated with higher HSPA1A expression, and smoking exposure was associated with elevated BAG3 levels among women, these observations do not establish causality or demonstrate a direct neuroprotective mechanism. The proteins evaluated in this study were measured in peripheral blood and therefore represent systemic biomarkers rather than direct measures of central nervous system biology. Consequently, the observed associations should be viewed as circulating proteomic signatures that are consistent with pathways implicated in experimental models of proteostasis and stress-response regulation. Although these findings align with hypotheses proposing sex-specific biological responses to smoking exposure, they do not establish that the identified proteomic changes mediate the reduced risk of PD observed in epidemiologic studies. Future longitudinal investigations incorporating cerebrospinal fluid biomarkers, neuroimaging, genetic approaches, and clinical outcomes will be required to determine whether these peripheral signatures reflect biologically relevant processes within the central nervous system.

## Conclusions

This study identifies sex-specific associations between cumulative smoking exposure and circulating proteostatic biomarkers within the UK Biobank Olink Explore 3072 dataset. Female smokers exhibited higher HSPA1A and BAG3 levels, particularly in the setting of higher circulating estradiol concentrations. These findings provide population-scale evidence that smoking exposure is associated with differential regulation of peripheral stress-response and proteostasis pathways in women. However, because the study is observational and relies on blood-based biomarkers, the results should be interpreted as associations rather than evidence of causal neuroprotective mechanisms. Further longitudinal and mechanistic studies are needed to determine whether these circulating proteomic signatures are related to biological processes relevant to PD susceptibility.

## References

[1] Rose KN, Schwarzschild MA, Gomperts SN (2024). Clearing the smoke: what protects smokers from Parkinson's disease?. Mov Disord.

[2] Lehrer S, Rheinstein PH (2022). Constipation and cigarette smoking are independent influences for Parkinson's disease. Cureus.

[3] Eldjarn GH, Ferkingstad E, Lund SH (2023). Large-scale plasma proteomics comparisons through genetics and disease associations. Nature.

[4] Bycroft C, Freeman C, Petkova D (2018). The UK Biobank resource with deep phenotyping and genomic data. Nature.

[5] Wik L, Nordberg N, Broberg J (2021). Proximity extension assay in combination with next-generation sequencing for high-throughput proteome-wide analysis. Mol Cell Proteomics.

[6] Pandey G, Garcia RC, Das D (2026). Genetically encoded constitutive upregulation of β2 subunit containing neuronal nicotinic acetylcholine receptors is neuroprotective in female parkinsonian mice. J Neurosci.

[7] Quik M, O'Leary K, Tanner CM (2008). Nicotine and Parkinson's disease: implications for therapy. Mov Disord.

[8] Oertel WH, Müller HH, Unger MM (2023). Transdermal nicotine treatment and progression of early Parkinson's disease. NEJM Evid.

[9] Liang CH, Huang TW, Chiu WT, Chung CC, Hong CT (2025). Nicotine therapy for Parkinson's disease: a meta-analysis of randomized controlled trials. Biomedicines.

[10] Paradossi U, De Caterina AR, Trimarchi G (2024). The enigma of the 'smoker's paradox': results from a single-center registry of patients with STEMI undergoing primary percutaneous coronary intervention. Cardiovasc Revasc Med.

[11] Lin H, Koren SA, Cvetojevic G, Girardi P, Johnson GV (2022). The role of BAG3 in health and disease: a "magic bag of tricks". J Cell Biochem.

[12] Qu HQ, Feldman AM, Hakonarson H (2022). Genetics of BAG3: a paradigm for developing precision therapies for dilated cardiomyopathies. J Am Heart Assoc.

